# Study of the Interfacial Interaction Performance of Branched Bonding Agents and CL-20

**DOI:** 10.3390/ma12091402

**Published:** 2019-04-30

**Authors:** Yanmei Zhao, Xiaoqing Wang, Xiaomeng Li, Yunjun Luo, Guoping Li

**Affiliations:** School of Materials Science & Engineering, Beijing Institute of Technology, Beijing 100081, China; 2120161203@bit.edu.cn (Y.Z.); xm.lee@bit.edu.cn (X.L.); yjluo@bit.edu.cn (Y.L.)

**Keywords:** CL-20, interfacial interaction, bonding agents, high energy composites

## Abstract

Adding bonding agents to the formulation is an effective way to solve the dewetting of 2,4,6,8,10,12-hexanitro-2,4,6,8,10,12-hexaazaisowurtzitane (CL-20) in the binder matrix. For the design and selection of the structure of a bonding agent suitable for CL-20, a series of branched polyether bonding agents with terminal groups substituted by cyano, ester and hydroxyl functional groups were employed. Contact angle, Fourier transform infrared spectra (FTIR) and X-ray photoelectron spectroscopy (XPS) measurements were performed, and the results were compared with that of common bonding agent, the boron trifluoride triethanolamine complex (TEA·BF_3_). The results revealed that the nitramine reinforcement of the three polar groups to CL-20 was in the order cyano group > hydroxyl group > ester group. It is proposed that CBPE-10,10 (cyano-terminated branched polyether bonding agent) had the strongest interfacial interaction with CL-20. The adhesion work value was 105.37 mN⋅m^−1^ and the adhesion degree was 41.04%. The bonding properties of CBPEs/CL-20 were better than those of TEA·BF_3_/CL-20.

## 1. Introduction

It is well established that 2,4,6,8,10,12-hexanitro-2,4,6,8,10,12-hexaazaisowurtzitane (C_6_H_6_N_12_O_12_, CL-20) is one of the most important energetic materials. CL-20 has emerged as a powerful alternative to hexahydro-1,3,5-trinitro-1,3,5-triazine (RDX) and octahydro-1,3,5,7-tetranitro-1,3,5,7-tetrazocine (HMX) because of its superior oxygen balance, higher heat of formation, and higher density [1,2,3,4]. Composites based on nitramine such as RDX, HMX and CL-20, suffer from a problem known as dewetting, that is, the weak interfacial adhesion of nitramine crystals/binders may cause the binder to detach from the nitramine surface under stress, and thus induce further crack damages. Dewetting adversely affects the processability [5], sensitivity [6] and mechanical properties [7,8] of the composite propellants and plastic bonded explosives (PBXs). Therefore, it is crucial to strengthen the interfacial interaction between CL-20 and the binder for a wide application of CL-20. However, previous studies have shown that CL-20 is more difficult to wet than HMX [9,10,11], in other words, dewetting is a more serious problem in CL-20-containing high energetic composites.

Many studies have shown that the addition of bonding agents significantly enhances the interfacial adhesion interactions of the nitramine/binder, thus effectively raising the mechanical properties of the propellants [12,13]. Bonding agents contain functional groups that have a favorable affinity for nitramine crystals and can chemically bond to the binder matrix during the curing process [8]. Consequently, bonding agents play a vital role in improving the mechanical properties of high energetic composites. The commonly-used small molecule bonding agents are aziridine, alkanolamine, polyamine and their derivatives, etc. The boron trifluoride triethanolamine complex (TEA·BF_3_), an alcoholic amine bonding agent, is a frequently-used bonding agent in propellants. It has been demonstrated that TEA·BF_3_ had good interfacial interaction with CL-20, and it has been applied in hydroxyl-terminated polybutadiene (HTPB) propellant [14,15,16]. Kim firstly proposed neutral polymeric bonding agents (NPBAs) in the 1990s. NPBAs not only solved the problem of small molecule bonding agents easily dissolving in the polar plasticizer, but also increased the number of polar groups in the bonding agents. Kim confirmed that NPBAs with cyano, ester and hydroxyl groups had a high affinity for the HMX particles, and the addition of NPBAs to HMX-filled poly(ethylene glycol) (PEG) binder remarkably increased the strength of the propellant [12,17]. Since then, many types of macromolecular bonding agents have been synthesized and applied [18,19,20,21], following the design idea of NPBAs. Chen [22] synthesized a series of dodecylamine-based bonding agents with the end groups substituted with cyano, ester and hydroxyl groups, which showed a good affinity with RDX and enhanced the mechanical properties of the HTPB propellant. 

Although previous studies have shown that the bonding agents with cyano, ester or hydroxyl groups had significant nitramine reinforcement, barely any studies have reported on the bonding agent of CL-20. Moreover, the affinity of different polar group to CL-20 is virtually unexplored. In this paper, a series of branched polyether bonding agent, with terminal groups substituted by cyano, ester and hydroxyl functional groups, were used. Branched polymers, containing many modifiable terminal active sites [23,24], are suitable as bonding agents for the chemical modification of terminal groups into different polar groups in order to evaluate the effects of the type and number of polar groups on the interfacial interaction performance of the bonding agent/CL-20. This study aimed to demonstrate the affinity of different polar groups to CL-20, thus providing a basis for the design and selection of the structure of the bonding agent. The static contact angle, FTIR and XPS were utilized as test methods to demonstrate the interfacial interaction between the branched bonding agents and CL-20, and compared with that of boron trifluoride triethanolamine complex (TEA·BF_3_).

## 2. Materials and Methods 

### 2.1. Materials

CL-20 (γ-polymorph, 20 µm, white crystal) was obtained from Liaoning Qingyang Chemical Industry Co., Ltd., China and dried at 60 °C before use. The branched polyether bonding agents (cyano-terminated branched polyether (CBPEs) and branched polyether (BPEs)) were synthesized in our laboratory. The functionality of the groups and the molecular weight for branched polyether bonding agents are shown in Table 1. TEA·BF_3_ was supplied by Xi’an New Chemical Material Company, China. Ethanol (analytical grade) was obtained from Beijing Chemical Plant, China. Diiodomethane (analytical grade) was purchased from Shanghai Macklin Biochemical Co., Ltd., China. Formamide (analytical grade) was purchased from Tianjing Fuchen Chemical Reagent Co., Ltd., China. Ethylene glycol was purchased from Beijing Tongguang Fine Chemicals Company, China.

### 2.2. Preparation of CL-20 Coated with Bonding Agents

CL-20 (0.002 mol) was mixed with the bonding agent (0.001 mol) and dissolved in ethanol (10 mL). After being continually stirred for one hour at room temperature, the mixture was filtered, and the precipitate was washed with ethanol several times to remove the surface bonding agent. After this, the CL-20 samples coated with different bonding agents were kept in a dry oven at 60 °C for two days.

### 2.3. Characterization

Scanning electron microscopy (SEM) measurements were performed with a thermal field emission scanning electron microscope instrument (JSM-7610F, JEOL, Tokyo, Japan) at an operating voltage of 5 KV. An ultrathin conductive coating was deposited before it was analyzed.

The contact angle was measured using an OCA contact angle analyzer (Datephysics Co, Stuttgart, Germany). Diiodomethane, formamide, and ethylene glycol were selected as the test fluids. The drop volume was 1 µL and the drop flow was at medium speed.

Fourier transform infrared spectra were recorded (Nicolet FTIR-8700, Thermo, Waltham, Massachusetts, USA) with a wavenumber resolution of 4 cm^−1^ and a single average of 64 scans at room temperature. KBr pellets of the samples were used.

X-ray photoelectron spectroscopy of pure CL-20 and CL-20 coated with bonding agents were recorded with an ESCALAB 250Xi electron spectrometer (Thermo Fisher Scientific Co, Waltham, Massachusetts, USA). Monochromatic Al-Kα irradiation at 72 W (12 kV at 6 mA) was used for the target (1486.6 eV) and under a vacuum of less than 10^−6^ MPa. The beam spot size was 50 µm. Survey scans were recorded with a 1 eV step and 150 eV pass energy and the high-resolution regions were recorded with a 0.1 eV step and 20 eV pass energy. Before data analysis, the high-resolution measurements were charge corrected using the C 1s signal at 284.8 eV. The XPS-PEAK software was used to process the data, including curve smoothing, deconvolution, background subtraction, normalization and curve fitting. 

## 3. Results and Discussion

### 3.1. Morphology of CL-20 and CL-20 Coated with Bonding Agents

**Figure 1:**
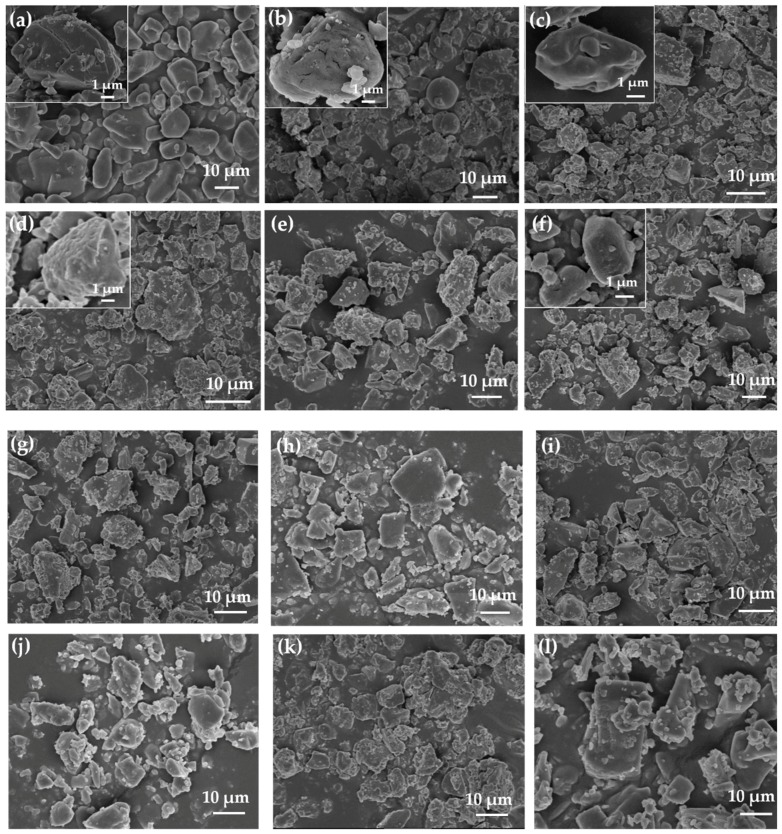
Scanning electron microscopy (SEM) pictures of (**a**) 2,4,6,8,10,12-hexanitro-2,4,6,8,10,12-hexaazaisowurtzitane (CL-20); (**b**) CBPE-3,3/CL-20; (**c**) CBPE-4,4/CL-20; (**d**) CBPE-5,5/CL-20; (**e**) CBPE-6,6/CL-20; (**f**) CBPE-8,8/CL-20; (**g**) CBPE-10,10/CL-20; (**h**) CBPE-3,6/CL-20; (**i**) CBPE-4,7/CL-20; (**j**) BPE-6/CL-20; (**k**) BPE-12/CL-20; (**l**) TEA·BF_3_/CL-20.

The SEM images of the CL-20 particles and CL-20 coated with bonding agents are shown in Figure 1. The surface of CL-20 became rough after coating with bonding agents (see Figure 1).

### 3.2. The Adhesion Work and Interfacial Tension between CL-20 and the Bonding Agents

Table 2 shows the contact angles (θ) of the reference liquid on the bonding agents. The values of contact angles less than 90° indicated that the reference liquids could wet the samples, and the closer the values were to 0°, the easier the samples were wetted. By comparing the values of the contact angles of CBPE-3,6/CBPE-6,6, CBPE-3,6/CBPE-3,3, and BPE-6/BPE-12, set out in Table 2, an increase in the number of cyano, ester and hydroxyl groups decreased the values of the contact angle of the bonding agents. Furthermore, the change in the number of cyano groups had the greatest influence on the value of the contact angle, which made it easier to improve the wettability of the bonding agents.

According to the formulas in Equations (1) and (2) [25], the surface tension ($\gamma_{s}$) of the bonding agents, polar component ($\gamma_{s}^{d}$) and nonpolar component ($\gamma_{s}^{p}$) [26] were calculated using the contact angles and are shown in Table 3.

(1) $$1+\cos\theta =2\left[\frac{{\left(\gamma_{s}^{d}\right)}^{1/2}{\left(\gamma_{l}^{d}\right)}^{1/2}}{\gamma_{l}}+\frac{{\left(\gamma_{s}^{p}\right)}^{1/2}{\left(\gamma_{l}^{p}\right)}^{1/2}}{\gamma_{l}}\right]$$

(2) $$\gamma_{s}=\gamma_{s}^{p}+\gamma_{s}^{d}$$

where $\gamma_{l}$, $\gamma_{l}^{d}$ and $\gamma_{l}^{p}$ are the surface tension, nonpolar part, and polar part of surface tension for test fluids, respectively, according to [27].

By comparing the surface properties of CBPE-3,3 with CBPE-3,6 according to Table 3, we found that the increase in the number of ester groups decreased the polar component of the surface tension and increased the non-polar component of the surface tension, thus decreasing the surface tension overall. Similarly, the surface properties of CBPE-3,6 and CBPE-6,6 were compared, as well as BPE-6 and BPE-12. It was indicated that with the increase in the number of cyano and hydroxyl groups, the polar and non-polar components of the surface tension were increased by 43% and 25%, respectively. The changes in the number of cyano, ester and hydroxyl groups affected the surface properties of the bonding agents. In addition, the surface properties of the components in high energy composites should be matched to optimize the interface properties. It was concluded that the surface properties of the branched bonding agents could be adjusted to be more compatible in composites by changing the number of three polar groups. The values of the interfacial tension ($\gamma_{AB}$) and adhesion work ($W_{AB}$) between the bonding agents and CL-20 were obtained using the formulas in Equations (3) and (4) [28,29] and are shown in Table 4.

(3) $$\gamma_{AB}=[{\left(\gamma_{A}^{d}\right)}^{1/2}-{\left(\gamma_{B}^{d}{)}^{1/2}\right]}^{2}+[{\left(\gamma_{A}^{p}\right)}^{1/2}-{\left(\gamma_{B}^{p}{)}^{1/2}\right]}^{2}$$

(4) $$W_{AB}=\gamma_{A}+\gamma_{B}-\gamma_{AB}$$

where $\gamma_{A}$, $\gamma_{A}^{d}$ and $\gamma_{A}^{p}$ are the surface tension, nonpolar part and polar part of the surface tension for bonding agents. $\gamma_{B}$, $\gamma_{B}^{d}$ and $\gamma_{B}^{p}$ are the surface tension, nonpolar part and polar part of the surface tension for CL-20. As shown in Table 4, the values of the interfacial tension of CBPEs/CL-20 and BPEs/CL-20 were lower than that of the TEA·BF_3_/CL-20, which indicated the better wettability of the CBPEs and BPEs.

The values of the adhesion work can be used to study the interfacial bonding strength [30]. The higher the value of the adhesion work, the stronger the interfacial interaction. As shown in Table 4, the values of the adhesion work of the CBPEs/CL-20 were higher than those of the TEA·BF_3_/CL-20 and increased with the increasing number of polar groups (cyano and ester groups). The maximum adhesion work was CBPE-10,10/CL-20, with a value of 105.37 mN·m^−1^. By varying the number of each kind of polar group in the bonding agents, the affinity between these polar groups to CL-20 can be accessed by comparing the change in adhesion work. The number of ester groups in CBPE-3,6 was doubled compared with CBPE-3,3, and the adhesion work of CBPE-3,6/CL-20 increased by 3% compared with CBPE-3,3/CL-20. Similarly, the number of cyano groups was doubled, and the adhesion work of CBPE-6,6/CL-20 increased by 18% compared with that of CBPE-3,6/CL-20. The adhesion work increased by 8% by the doubling of the hydroxyl number (comparing BPE-12/CL-20 with BPE-6/CL-20). It was concluded that the affinity between the three polar groups and CL-20 was in the order cyano group > hydroxyl group > ester group. Although the interfacial bonding strength between the BPEs and CL-20 could be improved by increasing the number of hydroxyl groups, including an excessive amount of hydroxyl groups in the propellant formulation could lead to the over-crosslinking of bonding agents with the binder, which could adversely affect the mechanical properties of the propellant [31]. Therefore, we focused on the interfacial interactions of the cyano and ester groups with CL-20 in the following sections.

### 3.3. Interaction between CL-20 and the Bonding Agents Studied by Fourier Transform Infrared Spectroscopy (FTIR)

**Figure 2:**
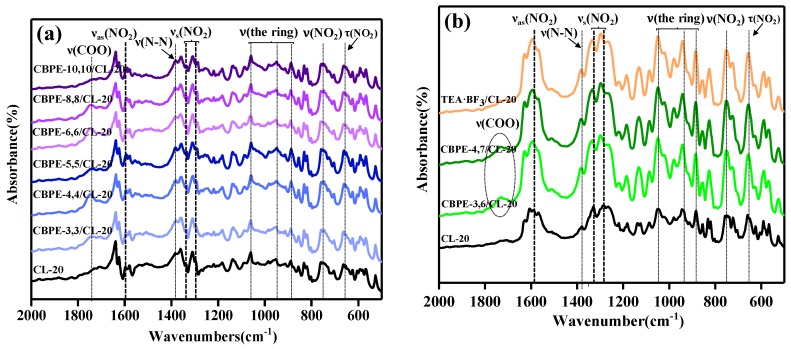
Fourier Transform Infrared Spectroscopy (FTIR) spectra of CL-20 and CL-20 coated with bonding agents: (**a**) CL-20 coated with CBPEs; (**b**) CL-20 coated with CBPE-3,6, CBPE-4,7 and TEA·BF_3_.

The position changes of the characteristic peaks in the infrared spectra can sensitively characterize the interfacial interaction between two phases [20]. FTIR was chosen to investigate the interaction between the bonding agents and CL-20. The FTIR spectra of CL-20 and CL-20 coated with bonding agents are shown in Figure 2, and the assignment of the characteristic peaks are listed in Table 5. 

In the FTIR spectrum of CL-20, 651 cm^−1^ and 751 cm^−1^ were assigned to the bending and stretching vibration peak of -NO_2_, whereas the signal at 883 cm^−1^, 943 cm^−1^ and 1049 cm^−1^ were assigned as the stretching modes of the ring [32]. The signal at 1383 cm^−1^ was the stretching vibration peak of N-N [13]. 1295 cm^−1^ and 1328 cm^−1^ were assigned as symmetric stretching modes ($\nu_{s}$) of -NO_2_, while 1590cm^−1^ was assigned as the asymmetric stretching modes ($\nu_{as}$) of -NO_2_ [33]. As shown in Figure 2, in the new absorption bands, 1749 cm^−1^ was assigned to C=O [34], which indicated that the CL-20 was successfully coated with CBPEs.

Spectral changes were observed for the samples of CBPEs/CL-20. The symmetric stretching and asymmetric stretching peaks of the -NO_2_ in CL-20 shifted to lower wavenumbers after being coated with CBPEs containing the same number of cyano and ester groups, yet shifted to higher wavenumbers after being coated with CBPE-3,6, CBPE-4,7 and TEA·BF_3_. The nitro groups, as an active group in CL-20, could hydrogen bond with the hydroxyl groups in the bonding agents leading to the shift towards a lower wavenumber. Aside from this, -CN induced -NO_2_ and the O acted as an electron acceptor, because the electronegativity of the N in -CN was lower than that of O in -NO_2_, causing the symmetric and asymmetric stretching peaks of -NO_2_ to shift towards lower wavenumbers [35]. However, the ester groups in CBPEs and fluorine atoms in TEA·BF_3_ enabled the induction of -NO_2_ and the N played a role of the electron donor, which resulted in the stretching vibration of -NO_2_ shifting to higher wavenumbers. It was considered that the variation was decidedly attributed to the combined action of hydrogen bonding, electronic effects and induction effects. 

**Figure 3:**
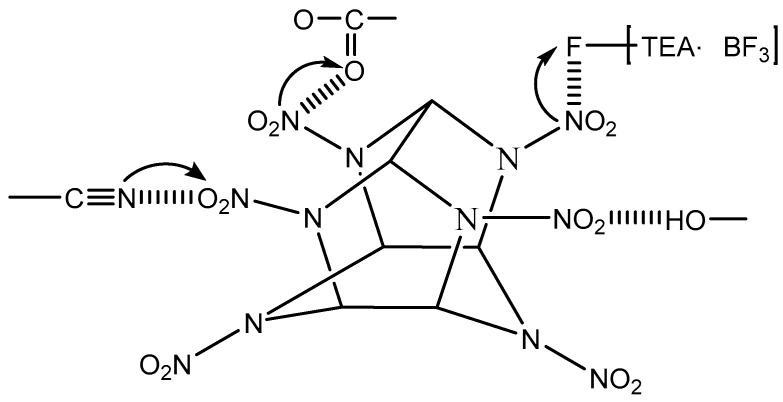
Interaction between CL-20 and polar groups.

The extent of shift of the nitro absorption peaks can indicate the strength of interaction between the polar groups and the nitro groups. The symmetric stretching and asymmetric stretching vibration peaks of –NO_2_ in CBPE-6,6/CL-20 had a shift of 10 cm^−1^, 7 cm^−1^ and 6 cm^−1^, respectively, to lower wavenumbers compared to CL-20 coated by CBPE-3,6. However, CBPE-3,6/CL-20 had a shift of 6 cm^−1^, 5 cm^−1^ and 3 cm^−1^ to higher wavenumbers compared to CL-20 coated with CBPE-3,3. It was concluded from the extent of movement that the interaction between the cyano group and the nitro group was stronger than that of the ester group, agreeing with the results obtained using contact angle measurement. The schematic diagram of the hydrogen bonding effects, electronic effect and induction effects can be deduced, as shown in Figure 3 (the arrow indicates the direction in which the electronic cloud moves).

### 3.4. Interaction between CL-20 and the Bonding Agents Studied by X-ray Photoelectron Spectroscopy (XPS)

**Figure 4:**
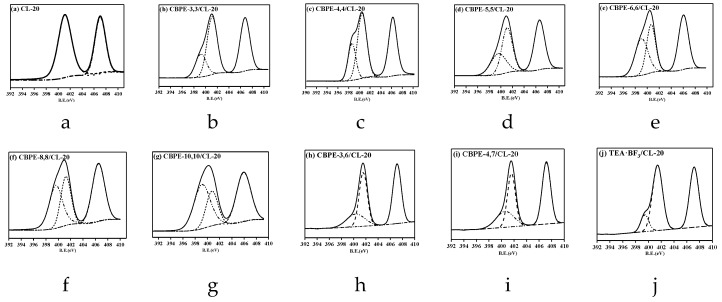
XPS N1s spectra fitting curves of pure CL-20 and CL-20 coated with bonding agents: (**a**) CL-20; (**b**) CBPE-3,3/CL-20; (**c**) CBPE-4,4/CL-20; (**d**) CBPE-5,5/CL-20; (**e**) CBPE-6,6/CL-20; (**f**) CBPE-8,8/CL-20; (**g**) CBPE-10,10/CL-20; (**h**) CBPE-3,6/CL-20; (**i**) CBPE-4,7/CL-20; (**j**) TEA·BF_3_/CL-20.

X-ray photoelectron spectroscopy was used to describe the interfacial interaction between CL-20 and the bonding agents. The adhesion performance between the bonding agents and CL-20 was characterized by analyzing the change in nitrogen content on the sample surface. The interfacial interaction between the bonding agents and CL-20 was analyzed by the change in the chemical shift of the nitrogen element. The energy spectrum peak of N1s was used to investigate the change in the nitrogen content, and the adhesion degree (R) was calculated using the formula in Equation (5) [36]:

(5) $$R=\frac{A_{N1s}}{B_{N1s}}$$

where $A_{N1s}$ refers to the special nitrogen atom peak area in the bonding agent coated on the surface of CL-20 in a narrow scan, and $B_{N1s}$ refers to the N1s total peak area of CL-20. The results are summarized in Table 6. The fitted N 1s spectra of CL-20 and CL-20 coated with bonding agents are all shown in Figure 4. The data were processed using the XPS-PEAK software, which utilized a least square approximation method, which maintained the peak position of the same bond as close as possible.

As shown in Table 6, the peaks at 401.95 eV and 407.10 eV were assigned to the -N< and -NO_2_ in pure CL-20 [37]. The peaks in the range 399~400 eV were assigned to –CN in the CBPEs and tertiary nitrogen in the TEA·BF_3_ [22].

The emission peaks of tertiary nitrogen (401.95 eV) in CL-20 shifted to a lower binding energy after coating with CBPEs. It was found that the emission peaks of the nitro group for CBPE-3,6/CL-20 increased by 0.39 eV compared with CBPE-3,3/CL-20, because the electron-withdrawing effect of ester groups on the nitro group was greater than the induction effect of the cyano group. Therefore, the decrease in the electron cloud density for –NO_2_ increased the binding energy. On the contrary, the binding energy of the nitro group for CBPE-6,6/CL-20 decreased by 0.64 eV compared to CBPE-3,6/CL-20 due to the domination by the induction effect of the cyano groups. According to the changes in the binding energy, it was considered that the affinity of the cyano groups to the nitro groups was stronger than that of the ester groups, which was consistent with the results of the contact angle and FTIR spectroscopy. The strong electronegativity of the fluorine atom in TEA·BF_3_ resulted in the increased binding energy of the nitro group by 0.08 eV compared to that of pure CL-20. The value of the adhesion degree for CBPE-10,10/CL-20 was the highest shown in Table 6 at 41.04%.

## 4. Conclusions

In this work, the interfacial interaction performance between the bonding agents (CBPEs, BPEs and TEA·BF_3_) and CL-20 particles was studied. The SEM images show that CL-20 particles were coated with bonding agents. The results of the interfacial tension measurement indicated that the CBPEs and BPEs showed a higher ability to wet CL-20 than TEA·BF_3_. The affinities of the polar groups to CL-20 were in the order–CN > -OH > -COO. Furthermore, the change in the number of cyano groups had the greatest influence on the reduction of the values of the contact angle, which makes it easier to improve the wettability of the bonding agents. According to the FTIR and XPS results, the interaction mainly originated from the interaction between the -NO_2_ and the polar groups in the bonding agents, and the interaction between the -CN and -NO_2_ was stronger than the interaction between the –COO and -NO_2_. The shift of the characteristic peak positions of -NO_2_ is attributed to hydrogen bonding, electronic effects and induction effects. The results of the interfacial characteristics suggest that the bonding agent CBPE-10,10 shows the strongest interfacial bonding interaction with CL-20, as the adhesion degree reaches 41.04%.

## Figures and Tables

**Table 1:** Parameters of the branched polyether bonding agents.

Sample	*f*^1^(-CN)	*f*(-COO)	*f*(-OH)	M_n_^2^(g·mol^−1^)
CBPE-3,3 ^3^	3	3	3	642
CBPE-4,4	4	4	2	709
CBPE-5,5	5	5	1	776
CBPE-6,6	6	6	6	1450
CBPE-8,8	8	8	4	1586
CBPE-10,10	10	10	2	1722
CBPE-3,6	3	6	3	684
CBPE-4,7	4	7	2	751
BPE-6 ^4^	0	0	6	441
BPE-12	0	0	12	1053

^1^ The functionality of the polar groups in cyano-terminated branched polyether bonding agents (CBPEs) and branched polyether bonding agents (BPEs). ^2^ Number average molecular weight of CBPEs and BPEs. ^3^ The first number and the second number separately represent the functionality of the cyano and ester groups in CBPEs. ^4^ The number represents the functionality of hydroxyl in BPEs.

**Table 2:** Contact angles (θ) of the reference liquids on the bonding agents and CL-20.

Sample	θ (°)		
	Diiodomethane	Formamide	Ethylene Glycol
CL-20	26.9	34.3	43.4
CBPE-3,3	66.3	58.1	48.7
CBPE-4,4	58.3	49.4	40.6
CBPE-5,5	50.3	40.2	35.2
CBPE-6,6	40.1	36.5	30.0
CBPE-8,8	20.1	19.7	18.2
CBPE-10,10	10.5	12.9	10.2
CBPE-3,6	58.9	60.2	59.1
CBPE-4,7	50.3	54.1	50.3
BPE-6	68.1	73.0	59.0
BPE-12	65.3	55.6	49.2
TEA·BF_3_	68.5	53.4	49.8

**Table 3:** Surface tensions of the bonding agents and CL-20.

Sample	$\gamma_{s}^{p}(\mathrm{mN}\cdot m^{-1})$	$\gamma_{s}^{d}(\mathrm{mN}\cdot m^{-1})$	$\gamma_{s}(\mathrm{mN}\cdot m^{-1})$
CL-20	2.04	40.61	42.65
CBPE-3,3	9.00	25.00	34.00
CBPE-4,4	9.49	29.70	39.19
CBPE-5,5	9.12	34.46	43.58
CBPE-6,6	7.34	39.82	47.16
CBPE-8,8	6.35	48.30	54.65
CBPE-10,10	4.54	60.68	65.22
CBPE-3,6	3.53	29.49	33.02
CBPE-4,7	3.88	34.34	38.22
BPE-6	4.20	23.62	27.82
BPE-12	9.00	25.70	34.70
TEA·BF_3_	10.76	24.01	34.77

**Table 4:** Interfacial tensions and adhesion works of the bonding agents/CL-20.

Sample	$\gamma_{AB}(\mathrm{mN}\cdot m^{-1})$	$W_{AB}(\mathrm{mN}\cdot m^{-1})$
CBPE-3,3/CL-20	4.35	72.30
CBPE-4,4/CL-20	3.58	78.26
CBPE-5,5/CL-20	2.79	83.44
CBPE-6,6/CL-20	1.65	88.16
CBPE-8,8/CL-20	1.53	95.78
CBPE-10,10/CL-20	2.50	105.37
CBPE-3,6/CL-20	1.09	74.58
CBPE-4,7/CL-20	0.56	80.31
BPE-6/CL-20	2.67	67.80
BPE-12/CL-20	4.17	73.19
TEA·BF_3_/CL-20	5.60	71.82

**Table 5:** Characteristics of CL-20 and CL-20 coated with bonding agents in the FTIR spectra.

Sample	τ (NO_2_)	ν(NO_2_)	ν(the ring)	ν(N-N)	$\nu_{s}({\mathrm{NO}}_{2})$	$\nu_{as}({\mathrm{NO}}_{2})$
CL-20	651	751	883,943,1049	1383	1295,1328	1590
CBPE-3,3/CL-20	651	751	883,943,1049	1383	1291,1325	1588
CBPE-4,4/CL-20	651	751	883,943,1049	1383	1287,1322	1586
CBPE-5,5/CL-20	651	751	883,943,1049	1383	1285,1320	1584
CBPE-6,6/CL-20	651	751	883,943,1049	1383	1281,1318	1582
CBPE-8,8/CL-20	651	751	883,943,1049	1383	1280,1317	1582
CBPE-10,10/CL-20	651	751	883,943,1049	1383	1278,1316	1581
CBPE-3,6/CL-20	651	751	883,943,1049	1383	1297,1330	1591
CBPE-4,7/CL-20	651	751	883,943,1049	1383	1298,1331	1592
TEA·BF_3_/CL-20	651	751	883,943,1049	1383	1295,1331	1592

**Table 6:** Specification and contents of the sample N1s spectra.

Sample	Attribution of N Atom	Binding Energy (eV)	Content (%)	R (%)
CL-20	-NO_2_	407.10	45.08	0
	-N<	401.95	54.92	
CBPE-3,3/CL-20	-NO_2_	406.80	38.21	18.51
	-N<	401.20	43.28	
	-CN	399.20	18.51	
CBPE-4,4/CL-20	-NO_2_	406.74	37.98	19.91
	-N<	401.11	42.10	
	-CN	398.62	19.91	
CBPE-5,5/CL-20	-NO_2_	406.64	39.13	24.19
	-N<	401.10	36.68	
	-CN	399.57	24.19	
CBPE-6,6/CL-20	-NO_2_	406.55	38.29	34.20
	-N<	400.70	31.76	
	-CN	399.10	34.20	
CBPE-8,8/CL-20	-NO_2_	406.50	38.44	34.86
	-N<	401.23	26.70	
	-CN	399.60	34.86	
CBPE-10,10/CL-20	-NO_2_	406.00	37.27	41.04
	-N<	400.74	21.69	
	-CN	399.20	41.04	
CBPE-3,6/CL-20	-NO_2_	407.19	37.68	19.78
	-N<	401.65	42.53	
	-CN	400.15	19.78	
CBPE-4,7/CL-20	-NO_2_	407.22	34.37	22.95
	-N<	401.57	42.67	
	-CN	400.12	22.95	
TEA·BF_3_/CL-20	-NO_2_	407.18	40.84	10.21
	-N<	401.43	48.94	
	-BF_3_-N-	399.51	10.21	
